# Systematic review and meta-analysis estimating association of cysticercosis and neurocysticercosis with epilepsy

**DOI:** 10.1371/journal.pntd.0005153

**Published:** 2017-03-07

**Authors:** Gabrielle Debacq, Luz M. Moyano, Héctor H. Garcia, Farid Boumediene, Benoit Marin, Edgard B. Ngoungou, Pierre-Marie Preux

**Affiliations:** 1 INSERM, Univ. Limoges, CHU Limoges, UMR_S 1094, Tropical Neuroepidemiology, Institute of Neuroepidemiology and Tropical Neurology, CNRS FR 3503 GEIST, Limoges, France; 2 Center for Global Health, Universidad Peruana Cayetano Heredia, Lima, Perú; 3 Epidemiology Unit. Hospital Regional II-2 Tumbes, Perú; 4 Instituto Nacional de Ciencias Neurológicas. Unidad de Cisticercosis. Lima, Perú; 5 Université des Sciences de la Santé, EA NEMIT, Faculté de Médecine, Libreville, Gabon; 6 CHU, CEBIMER, Limoges, France; Universidad Nacional Autónoma de México, MEXICO

## Abstract

**Background:**

We reviewed studies that analyzed cysticercosis (CC), neurocysticercosis (NCC) and epilepsy across Latin America, Asia and Sub-Saharan Africa, to estimate the odds ratio and etiologic fraction of epilepsy due to CC in tropical regions.

**Methodology:**

We conducted a systematic review of the literature on cysticercosis and epilepsy in the tropics, collecting data from case-control and cross-sectional studies. Exposure criteria for CC included one or more of the following: serum ELISA or EITB positivity, presence of subcutaneous cysts (both not verified and unverified by histology), histology consistent with calcified cysts, and brain CT scan consistent with NCC. A common odds-ratio was then estimated using meta-analysis.

**Principal findings:**

37 studies from 23 countries were included (n = 24,646 subjects, 14,934 with epilepsy and 9,712 without epilepsy). Of these, 29 were case-control (14 matched). The association between CC and epilepsy was significant in 19 scientific articles. Odds ratios ranged from 0.2 to 25.4 (*a posteriori* power 4.5–100%) and the common odds ratio was 2.7 (95% CI 2.1–3.6, p <0.001). Three subgroup analyses performed gave odds ratios as: 2.2 (EITB-based studies), 3.2 (CT-based studies), 1.9 (neurologist-confirmed epilepsy; door-to-door survey and at least one matched control per case). Etiologic fraction was estimated to be 63% in the exposed group among the population.

**Significance:**

Despite differences in findings, this meta-analysis suggests that cysticercosis is a significant contributor to late-onset epilepsy in tropical regions around the world, and its impact may vary depending on transmission intensity.

## Author summary

Cysticercosis is a helminthic infection of the central nervous system (CNS) and the leading cause of late onset epilepsy in low-and middle-income countries. This neurological disease is a public health problem in Sub-Saharan Africa, Asia and Latin America, affecting impoverished rural and peri-urban populations where sanitation is inadequate. Diagnostic criteria for NCC vary according to regional availability and access to imaging and serological tests. This systematic review highlights the lack of appropriate methodology in most observational studies, with few studies including control groups, a basic epidemiological criteria needed to demonstrate an association. EITB for cysticercosis was widely used to measure exposure, and brain CT scan was a non-invasive alternative used to identify cysts. Neurocysticercosis (NCC) is a preventable neurological condition in the tropics despite resource limitations in LMIC regions. Well-designed studies are needed to provide quality evidence to support control interventions and surveillance systems for this important zoonotic disease.

## Introduction

Cysticercosis (CC) is a parasitic infection caused by the larva stage (c*ysticercus)* of the tapeworm *Taenia solium*. It has been a major public health problem since historical times [1], and remains so, particularly in the developing world (low-and middle-income countries; LMIC), due to inadequate hygiene, rudimentary pig management and slaughter, and poor waste water management [2]. Developed regions such as Europe and North America are considered to be virtually free of endemic transmission, although there remains a substantial disease burden in these regions due to migration [3]. Neurocysticercosis (NCC) is considered a common helminthic infection of the central nervous system (CNS) across Latin America, Sub-Saharan Africa and Asia [4–7], and a common cause of late-onset epilepsy in LMIC [4,5,8]. For instance, a study in Burundi showed a strong link between CC and epilepsy, with an etiologic fraction of 50% (95% CI: 42–57) and an odds ratio of 3.8 (95% CI: 2.5 to 5.1) [6]. It was estimated in a recent meta-analysis that people infected with CC in Sub-Saharan Africa (SSA) are at 3.4–3.8 fold greater risk of having epilepsy [7]. It is noted that despite the importance of these diseases at an individual and population level, there are still discrepancies in the literature about their precise impact [4]. Moreover, earlier reviews focused on specific regions alone [7]. We conducted a review of studies that analyzed CC, NCC, and epilepsy across Latin America, Asia and Africa, to estimate the probability and etiologic fraction of epilepsy due to CC in tropical regions.

## Methods

### Literature search

Systematic searches were conducted for articles in English and French using the following databases: Medline, Scopus, Science Direct, Ingenta Connect, Refdoc (formerly Article Science). We also searched for articles and theses in the bibliographic database of the Institut d’Epidemiologie et de Neurologie Tropicale http://www.unilim.fr/ient/. Keywords used were (cysticercosis OR *Taenia solium* OR neurocysticercosis) AND epilepsy. Logical operators (AND, OR, NOT) were used. Bibliographies of published reviews and meta-analyses were also searched.

#### Data extraction

Two reviewers (GD and PMP) extracted data using methodology applied in previous meta-analyses [7] that focused on sub-Saharan Africa. Data types collected included; General: authors, year of publication, country, and study design used. Epilepsy: case sources, definition used, how and who confirmed epilepsy, source of people without epilepsy i.e. controls, and matching criteria. *CC*: methods used to evaluate CC and NCC (serological tests including enzyme-linked immunoelectrotransfer blot-EITB and enzyme-linked immunosorbent assay-ELISA, as well as neuroimaging) Methods: sample size for the following four groups: people with epilepsy affected by cysticercosis (PWE + CC), people with epilepsy not affected by cysticercosis (PWE—CC), people without epilepsy affected by cysticercosis (PWOE + CC), people without epilepsy unaffected by cysticercosis (PWOE—CC).

Eligible studies included those that 1) had epilepsy as a disease of interest and cysticercosis as exposure, 2) estimated sample size using appropriate techniques, 3) included detailed methods for diagnosis and determination of exposure, and 4) included a **control group in the analysis**. Case-reports, notes, letters to the editor, scientific reviews and other meta-analyses were excluded at this stage. For manuscripts in which authors presented results for multiple methods, we followed an order of priority. For instance, we considered computed tomography (CT) results more relevant than EITB or ELISA assays, and we retained EITB in preference to ELISA.

### Subgroup analyses

We conducted three subgroup analyses by taking into account those studies that used specific diagnostic tools for CC or NCC and epilepsy. The first group comprised studies that used EITB to determine CC exposure. The second analysis included studies that used brain CT scan to assess NCC exposure. The third analysis involved studies that had used standardized diagnostic methods to confirm epilepsy in population-based studies, such as neurological surveys applied in a door-to-door fashion with evaluation by well-trained general practitioners and /or neurological evaluation to confirm cases, and including at least one matched control per case. Finally, we performed an analysis by continent.

### Statistical analysis

For each of the selected studies, the odds ratio (OR) and its 95% confidence interval was determined using Epi-Info 6 (Centers for Disease Control and Prevention, Atlanta, USA). A meta-analysis was used to estimate the risk of developing epilepsy when exposed to CC, applying a random-effects model using Stata software, version 10.1 (Stata-Corp, College Station, TX, USA) to account for the variance of each included study [9]. Odds ratios (OR) and 95% confidence intervals (95% CIs) were determined. Homogeneity was assessed by I squared tests. Subgroup analyses were also conducted for studies ascertaining CC by EITB assays, CT scan and those studies that followed certain requirements for determining epilepsy (as mentioned under Methods, above). Because epilepsy has multiple causes and associated factors, we calculated the etiologic fraction (EF) i.e. the proportion of cases “attributable” to cysticercosis, by comparing the prevalence among exposed and the unexposed. The EF provided an unadjusted estimate of the proportion of cases of epilepsy that could be prevented if exposure to CC were eliminated. The etiologic fraction was based on the pooled estimate of risk, rather than single risk estimates for individual studies, by using the following formula: proportion exposed (common OR-1)/proportion exposed (common OR-1) +1.

## Results

**Fig 1:**
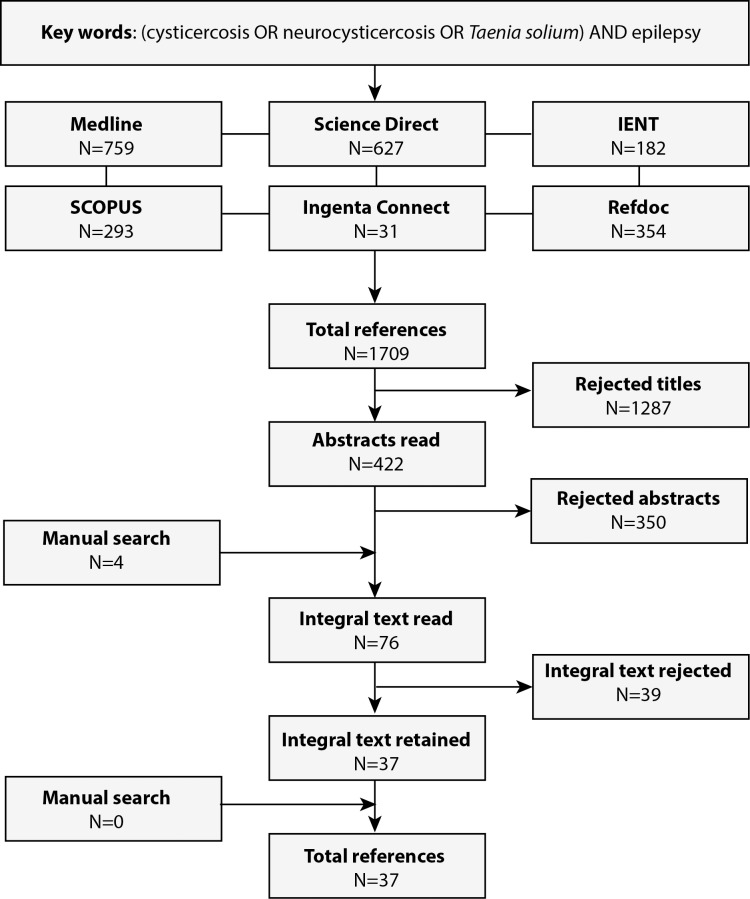
Flowchart of literature search.

**Fig 2:**
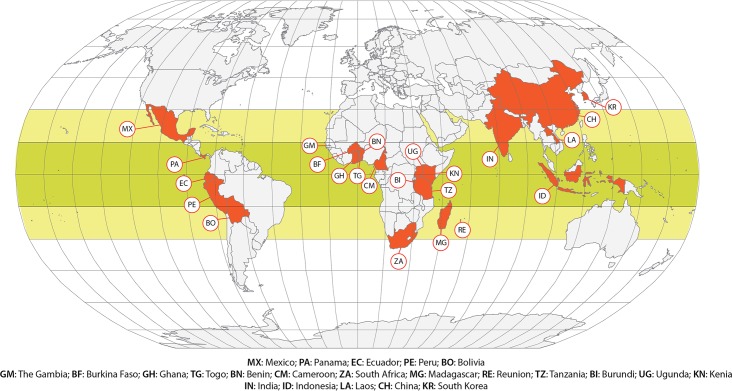
Locations of studies that evaluated association of cysticercosis and epilepsy.

In total, our searches identified 1709 publications. Of these, 1287 articles were excluded at the title level and 350 at the abstract level because they did not meet the inclusion criteria. Seventy-six articles were read in entirety; 37 were found to meet inclusion criteria and were included in the analysis (see Fig 1). These 37 studies (see Fig 2) were conducted in 23 different countries (five countries each from Asia and Latin America, and 13 from Africa). In total, these 37 studies covered 24,646 subjects (14,934 PWOE and 9,712 PWE). Seventy eight percent (29/37) were case-control studies, of which 14/29 (48.27%) were matched studies, (see Table 1).

**Table 1:** Description of the methods used in studies seeking an association between cysticercosis and epilepsy, classified by year of publication.

					People with epilepsy			People without epilepsy		Exposure		
Authors (year)	Country	Continent	Sub-continent	Design	Sources	Definition	Conf	Sources	criteria	Examination	Criteria/CC	Criteria/NCC
Chopra, 1981 [22]	India	Asia	Southern Asia	CCS	Hospital	NS	NS	GenPop	None	Cranial X-Ray, HAT	(+) HAT	(+)Cranial X-ray
Maldonado, 1986 [11]	La Réunion	Africa	Eastern Africa	CCS	Hospital	NS	NS	Hospital	None	X-Ray st	Calcified Lesion	None
Mignard, 1986 [23]	La Réunion	Africa	Eastern Africa	CCS	Hospital	NS	CK	Hospital	None	ELISA, CT	(+) ELISA	(+) CT
Dumas, 1989 [24]	Togo	Africa	Western Africa	CSS	GenPop	NS	Neurol	GenPop	House ^a^	Bx, Cranial X-ray, ELISA	(+) ELISA/His	(+) Cranial X-ray
Gracia, 1990 [25]	Panama	America	Central America	CCS	GenPop	ILAE 1970	Neurol	GenPop	sex, age >5	WB	(+)WB	None
Dansey, 1992 [26]	South Africa	Africa	South Africa	CCS	Miners	NS	CE	Miners	None	CT	None	(+) CT
Nzisabira, 1992 [27]	Burundi	Africa	Eastern Africa	CCS	GenPop	NS	Neurol	GenPop	None	ELISA (CSF), CT,Bx	(+)ELISA	(+)ELISA/(+) CT
Sarti, 1992 [28]	Mexico	America	North America	CSS	GenPop	NS	NS	GenPop	None	EITB	(+)EITB	None
García, 1993 [29]	Peru	America	South America	CCS	Hospital	NS	Neurol	Hospital	None	EITB	(+)EITB	None
Kong, 1993 [30]	Korea	Asia	Eastern Asia	CCS	GenPop*	NS	NS	GenPop	None	ELISA	(+)ELISA	None
Bouteille, 1994 [31]	Benin	Africa	Western Africa	CSS	GenPop ^a^	ILAE 1993	Neurol	GenPop	None	Bx, ELISA	(+)ELISA/His	None
Theis, 1994 [32]	Indonesia	Asia	South-Eastern Asia	CCS	Hospital	NS	NS	GenPop	None	ELISA, EITB	(+) Elisa/EITB	None
Aranda-Alvarez, 1995 [33]	Mexico	America	North America	CSS	GenPop ^b^	NS	NS	GenPop ^b^	None	ELISA-Ag	(+)ELISA-Ag	None
Grill, 1996 [34]	Madagascar	Africa	Eastern Africa	CCS	Hospital ^a^	NS	Neurol	Hospital	None	CT, ELISA /EITB (CSF)	(+) ELISA/EITB	(+) ELISA /EITB /CT
Andriantsimahavandy, 1997 [8]	Madagascar	Africa	Eastern Africa	CCS	Hospital	OMS 1981	CK	Hospital	Province, sex, age>10	EITB (CSF/ser)	(+)EITB	(+)EITB (CSF)
Handali, 1997 [35]	Indonesia	Asia	South-Eastern Asia	CSS	GenPop	NS	NS	GenPop	None	Bx	Cyst presence	None
Newell, 1997 [12]	Burundi	Africa	Eastern Africa	CCS	MR	NE	MD	Family	Household	ELISA-Ag, EITB	(+)ELISA-Ag/EITB	None
Correa, 1999 [36]	Mexico	America	North America	CCS	GenPop	NS	NS	GenPop	None	ELISA-Ag, EITB	(+)ELISA-Ag/EITB	None
Cruz, 1999 [37]	Ecuador	America	South America	CCS	GenPop	ILAE 1993	Neurol	GenPop	None	EITB, CT	(+)EITB	(+) CT
Balogou, 2000 [38]	Togo	Africa	Western Africa	CSS	GenPop	ILAE 1993	Neurol	GenPop	None	Bx, Cranial X-ray, ELISA	(+) ELISA/His	(+) Cranial X-ray
Mittal, 2001 [39]	India	Asia	Southern Asia	CCS	Hospital	NS	NS	NE	None	ELISA	(+)ELISA	None
Nicoletti, 2002 [13]	Bolivia	America	South America	CCS	GenPop	ILAE 1993	Neurol	GenPop	Villa, sex, age >5	EITB	(+)EITB	None
Macharia, 2002 [40]	Kenya	Africa	Eastern Africa	CCS	Hospital	ILAE 1993	CK	Hospital	Province,age,sex	ELISA	(+)ELISA	None
Rakatobe, 2002 [41]	Madagascar	Africa	Eastern Africa	CCS	GenPop	ILAE 1989	Neurol	GenPop	Family	ELISA, WB	(+) WB	None
Nsengiyumva, 2003 [6]	Burundi	Africa	Eastern Africa	CCS	Hospital	ILAE 1993	Neurol	Hospital	Province,age	ELISA	(+)ELISA	None
Dongmo, 2004 [42]	Cameroun	Africa	Middle Africa	CCS	GenPop ^a^	ILAE 1993	Neurol	GenPop	Age > 5	ELISA	(+)ELISA	None
Del Brutto, 2005 [43]	Ecuador	America	South America	CCS	GenPop	ILAE 1989	Neurol	GenPop	Sex, age > 5	WB, CT	(+)WB	(+) CT
Montano, 2005 [44]	Peru	America	South America	CSS	GenPop	ILAE 1989	Neurol	GenPop	None	EITB, CT	(+)EITB	(+) CT
Li, 2006 [45]	China	Asia	Eastern Asia	CSS	GenPop	NS	MD	GenPop	None	ELISA	(+)ELISA	None
Tran, 2007 [46]	Laos	Asia	South-Eastern Asia	CCS	GenPop	ILAE 1993	Neurol	GenPop	Villa, sex, age > 5	ELISA, WB	(+) ELISA/WB	None
Prasad, 2008 [47]	India	Asia	Southern Asia	CCS	GenPop	ILAE 1993	NS	Family	Family	EITB, MRI	(+)EITB	(+) MRI
Winkler, 2009 [10]	Tanzanie	Africa	Eastern Africa	CCS	Hospital ^b^	Winkler 2007	Neurol	Hospital	None	ELISA (CSF,Ser), CT	(+)ELISA	(+)ELISA(CSF)/ CT
Secka, 2010 [48]	Gambia	Africa	Western Africa	CCS	Hospital, MR	ILAE 1989	NS	GenPop	Villa, sex, age > 5	ELISA-Ag, EITB, CT	(+)ELISA-Ag/EITB	(+) CT
Nitiéma, 2012 [49]	Burkina Faso	Africa	Western Africa	CCS	GenPop ^c^	ILAE 2006	MD	GenPop ^c^	None	ELISA-Ag	(+)ELISA-Ag	None
Singh, 2012 [50]	India	Asia	Southern Asia	CCS	GenPop ^a^	ILAE 1989	Neurol	GenPop ^a^	Sex, age > 2	EITB	(+)EITB	None
Elliott, 2013 [51]	Cameroun	Africa	Middle Africa	CCS	MR	ILAE 1989	MD	GenPop	Sex, age	EITB	(+)EITB	None
Ngugi, 2013 [52]	Kenya *	Africa	Eastern Africa	CCS	GenPop ^d^	ILAE 1989	MD	GenPop ^d^	Age	WB	(+)WB	None

Source, NS: non-specific, GenPop: general population, GP*: population from Charity centers, GenPop a: General population older than 5 years old, GenPop b: General Population older than 14 years old. GenPop c: General Population older than 7 years old, GenPop d: General Population followed by centers for surveillance of health and demographic, Hospital a: Hospital Population older than 1 year old, Hospital b: Hospital Population older than 5 years old, MR: Medical Records.

Criteria, House a: House & Neighborhood. Confirmation, CK: cases know from local health centers, CE: clinical evaluation, MD: medical doctor, Neurol: neurologist.

HAT: hemagglutination test, X-Ray st: X-Ray soft Tissue, WB: Western Blot, Bx: Biopsy, His: Histology, Elisa-Ag: Elisa Antigen, Ser: serum, CSF: Cerebro Spinal Fluid

CT: computed tomography of the brain, MRI: Magnetic Resonance of the Brain

Kenia

*: Kenya, Sud-Africa, Uganda, Tanzania, Ghana.

CSS: cross-sectional study, CCS: case-control studies, Conf: confirmation, Criteria: selection criteria

### Epilepsy, CC and NCC

Twenty studies defined epilepsy, of which 18/20 (90%) followed at least one definition recommended by the International League against Epilepsy (ILAE, 1981, 1989, 1993, 2006). One study each used definitions proposed by the World Health Organization and that recommended for LMICs [10]. As noted in **Table 1**, there was great variability in the tools used for assessing exposure to CC, ranging from physical examination of subcutaneous nodules to Computed Tomography of the brain (CT), MRI images, cyst histology, and bioassays in serum or cerebrospinal fluid (CSF).

A total of 21/37 (56.75%) studies determined exposure to CC by detecting antibodies or antigens in serum using ELISA or ELISA-Ag. Seven studies used EITB to confirm or refute the results of ELISA and 12 studies used only EITB to determine CC exposure. One study used a hemagglutination test with sheep red blood cells sensitized to cysticercus antigens to determine exposure to CC. NCC exposure was determined by measuring antibodies in the CSF, but only 5/37 studies (13.51%) did so, by using ELISA (n = 3) or EITB (n = 2). CT was used in 14 studies, including 13 to assess NCC, and one [11] focused on the soft parts of the thigh.

### Association between CC and epilepsy

As shown in **Table 2**, the association between CC and epilepsy was statistically significant in 19 studies, leaving 18 with a non-significant association. The association was in fact nearly significant for two studies [12,13]. Odds ratios ranged from 0.2 to 25.4 and the *a posteriori* statistical power ranged from 4.5% to 100.0%.

**Table 2:** Results obtained in studies looking for an association between cysticercosis and epilepsy classified by year of publication.

	PWE	PWOE	PWE CC+		PWOE CC+		SP		
Authors	n	n	n	%	n	%	(%)	OR (95%IC)	P value
Chopra, 1981 [22]	771	98	267	25.7	2	2	100	25.4 (6.2–104.0)	< 0.001
Maldonado, 1986 [11]	240	385	40	16.7	19	5	99.9	3.9 (2.2–6.8)	< 0.001
Mignard, 1986 [23]	242	385	45	18.6	32	8.3	96.9	2.5 (1.6–4.1)	< 0.001
Dumas, 1989 [24]	88	1439	27	30.7	98	6.8	100	6.1 (3.7–10.0)	< 0.001
Gracia, 1990 [25]	19	36	1*	5.3	1*	2.8	6.6	1.9 (0.1–32.9)	0.772
Dansey, 1992 [26]	165	138	63	38.2	20	14.5	99.5	3.6 (2.1–6.4)	< 0.001
Nzisabira, 1992 [27]	98	30	40	40.8	1*	3.3	96.9	20.0 (2.6–152.9)	< 0.001
Sarti, 1992 [28]	16	1533	5	31.3	162	10.6	67.8	3.9 (1.3–11.2)	0.245
Kong, 1993 [30]	189	309	22	11.6	8	2.6	98.6	1.9 (1.1–3.3)	0.018
Garcia, 1993 [29]	2667	750	108	4.1	16	2.1	66.4	5.0 (2.2–11.4)	< 0.001
Bouteille, 1994 [31]	22	1421	2	9.1	55	3.9	21.2	2.5 (0.6–10.9)	0.49
Theis, 1994 [32]	74	746	10	13.5	94	12.6	4.5	1.1 (0.5–2.2)	0.967
Aranda-Alvarez, 1995 [33]	46	854	3	6.5	6	0.7	92.7	9.9 (2.4–40.8)	0.002
Grill, 1996 [34]	256	113	153	59.8	30	26.5	100	4.1 (2.5–6.7)	< 0.001
Andriantsimahavandy, 1997 [8]	104	104	33	31.7	14	13.5	88.6	3.0 (1.5–6.0)	0.003
Handali, 1997 [35]	241	260	163	67.6	215	82.7	99.2	0.4 (0.3–0.7)	< 0.001
Newell, 1997 [12]	103	72	12	11.7	2	2.8	56.8	4.6 (1.0–21.3)	0.065
Cruz, 1999 [37]	26	118	14	53.8	17	14.4	99.4	7.0 (2.7–17.5)	< 0.001
Correa, 1999 [36]	68	133	15	22.1	17	12.8	38	1.9 (0.9–4.2)	0.134
Balogou, 2000 [38]	115	1343	12	10.4	37	2.8	99.1	4.1 (2.1–8.1)	< 0.001
Mittal, 2001 [39]	1881	50	196	10.4	1*	2	96.1	5.7 (0.8–41.5)	0.088
Nicoletti, 2002 [13]	113	233	22	19.5	27	11.6	47	1.8 (1.0–3.4)	0.071
Macharia, 2002 [40]	99	124	5	5.1	3	2.4	19.1	2.2 (0.5–9.2)	0.492
Rakatobe, 2002 [41]	99	107	2	2	13	12.1	72.4	0.2 (0.1–0.7)	0.011
Nsengiyumva, 2003 [6]	324	648	193	59.6	204	31.5	100	3.2 (2.4–4.2)	< 0.001
Dongmo, 2004 [42]	93	81	17	18.3	12	14.8	9.3	1.3 (0.6–2.9)	0.683
Del Brutto, 2005 [43]	19	19	5	26.3	1	5.3	42.8	6.4 (0.7–61.5)	0.182
Montano, 2005 [44]	39	111	15	38.5	26	23.4	41.8	2.0 (0.9–4.5)	0.109
Li, 2006 [45]	55	445	9	16.4	11	2.5	99.9	7.7 (3.0–19.6)	< 0.001
Tran, 2007 [46]	31	124	1*	3.2	6	4.8	5.2	0.7 (0.1–5.7)	0.923
Prasad, 2008 [47]	60	107	29	48.3	31	28.9	70.9	2.3 (1.2–4.4)	0.02
Winkler, 2009 [10]	212	198	38	17.9	10	5.1	98.2	4.1 (2.0–8.5)	< 0.001
Secka, 2010 [48]	210	420	1*	0.5	1*	0.2	6.6	2.0 (0.1–32.2)	0.802
Nitiéma, 2012 [49]	39	814	5	12.9	28	3.4	67.1	4.1 (1.5–11.4)	0.022
Singh, 2012 [50]	106	106	27	25.5	13	12	77.7	2.4 (1.2–5.1)	0.011
Elliott, 2013 [51]	249	245	11	4.4	13	53	7.6	0.8 (0.4–1.9)	0.803
Ngugi, 2013 [52]	533	835	15	28.1	18	21.5	52.7	1.3 (0.7–2.6)	0.533

*Result = 0 in the study, modified to calculate the odds ratio, otherwise OR independent, SP: a posteriori statistical power

OR: Odds ratio, PWE + CC: people with epilepsy affected by cysticercosis, PWE—CC: people with epilepsy not affected by cysticercosis, PWOE + CC: people without epilepsy affected by cysticercosis, PWOE–CC: people without epilepsy unaffected by cysticercosis

### Meta-analysis and subgroup analyses

**Fig 3:**
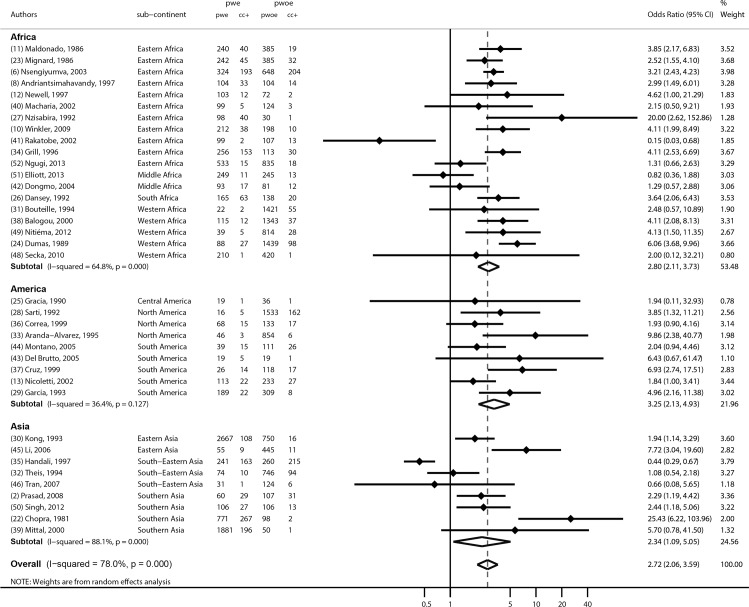
Meta-analysis assessing the association between CC and epilepsy globally and by continents: OR (odds ratio) of each study and common OR estimated using a random effects model

A meta-analysis of 37 studies based on the determination of exposure through detection of antibodies by ELISA or EITB, antigen detection by ELISA, or CT findings, is shown in Fig 3. The common odds ratio was estimated to be 2.7 (95% CI 2.1–3.6), p<0.001. Heterogeneity was substantial with a I squared at 78% (p<0.0001).

**Fig 4:**
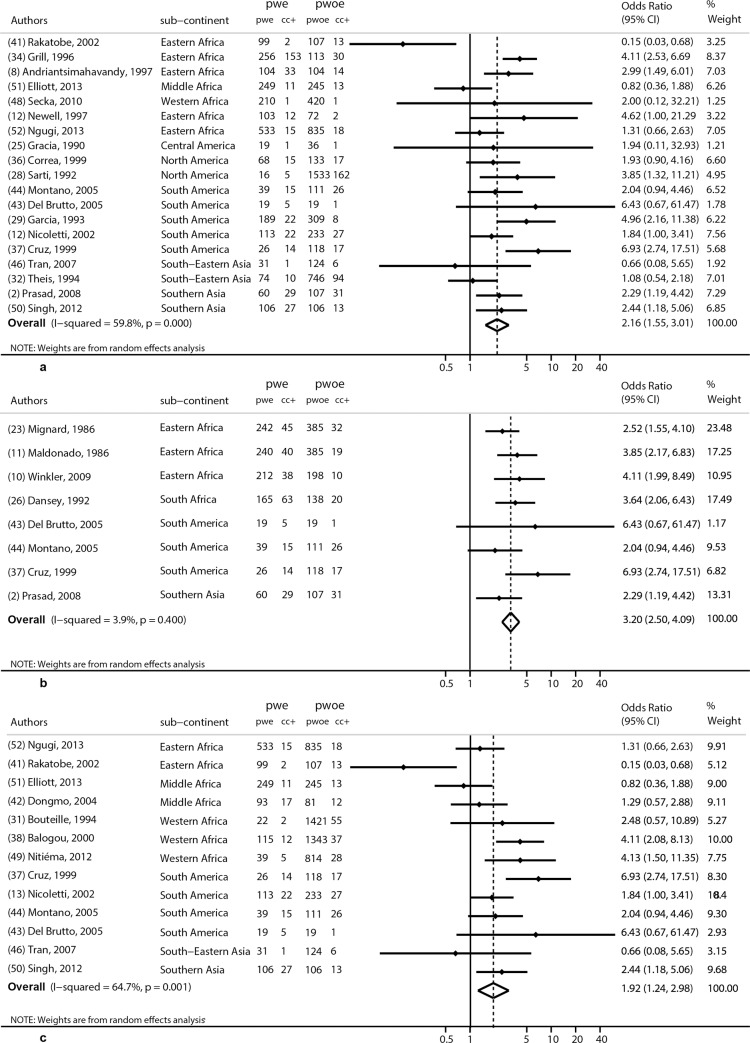
Various subgroup meta-analyses (EITB: Fig 4A, CT scan: Fig 4b; best studies: Fig 4c) assessing the association between CC and epilepsy in Latin America, Asia and Africa: OR (odds ratio) of each study and common OR estimated using a random effects model.

Three subgroup analyses were also performed as detailed in the methods section above. The first was based on studies that used EITB (n = 19), Fig 4A. The common odds ratio obtained was 2.2 (95% CI 1.6–3.0), p<0.001. Another subgroup analysis (Fig 4B) was based on studies that used brain computer tomography (n = 8). This gave a common odds-ratio of 3.2, (95% CI 2.5–4.1, p<0.001). The third subgroup analysis, Fig 4C, was based on the methods used to confirm epilepsy (n = 13), and gave a common odds ratio of 1.9 (95% CI 1.2–3.0), p<0.001. We also performed an analysis by continent, showing that the effect was quite similar around the world (Fig 3).

### Etiologic fraction

The etiologic fraction was estimated to be 63.0% (95% CI: 61.4–64.5) in the exposed group among the population. In other words, 63% of epilepsies were reportedly due to CC.

## Discussion

Our work was based on 37 studies conducted in many regions of Latin America, Asia and Africa. Particular efforts were made to identify studies by searching many databases and sources, including those that do not have a large international readership or were not in English. However, published information was available from only 23 countries, suggesting an evident information gap (see Fig 2).

A substantial proportion of these publications (n = 11) reported hospital-based studies, four were performed in health centers or medical clinics and another in a very specific population of mine-workers. There is a need to conduct well-designed interventions with appropriate methodology and to use validated tools to improve data quality, thereby reducing basic epidemiological biases. The lack of a control group, even in analytic cross-sectional studies, made it impossible to probe the association between this CNS helminthic infection and late-onset epilepsy by itself (i.e. ELISA or EITB for cysticercosis tested in PWE vs general population). This is one reason why several wide-scale or hospital studies were not included in this meta-analysis [14,15,16]. This type of study is also vulnerable to selection bias, particularly in rural areas, as epilepsy is stigmatized and may not also be visible (partial, mild seizures) or reported [17]. The remaining 21 studies were population-based that did include a control group.

The association between CC and epilepsy was statistically significant in only 19 studies, (Tables 1 and 2) and nearly significant in two studies. The odds ratios ranged from 0.2–25.4, and the *a posteriori* statistical power from 4.5% to 100.0% (Table 3). This wide variability could in part be due to non-adjustment of one or several other factors responsible for epilepsy occurrence. Many other factors, such as family predisposition, childbirth problems or head trauma, could lead to epilepsy and almost all studies failed to take into account all of these possible factors. Data elsewhere also support evidence that while in some populations there is a positive association between CC and epilepsy [12], in other studies conducted at a similar point of time these associations are absent [18]. Moreover, several studies with positive association between CC and epilepsy have their fair share of inconsistencies as well. For instance, one study in Burundi used an unmatched case-control study design [12] in which there were fewer control subjects than cases and controls were recruited from the same families as the cases.

**Table 3:** Results obtained in studies looking for an association between cysticercosis and epilepsy classified by continent and sub-continent.

			PWE	PWOE	PWE CC+		PWOE CC+		SP	
Authors	continent	sub-continent	n	n	n	%	n	%	(%)	OR (95%IC)
Chopra, 1981 [22]	Asia	Southern Asia	771	98	267	25.7	2	2	100	25.4 (6.2–104.0)
Maldonado, 1986 [11]	Africa	Eastern Africa	240	385	40	16.7	19	5	99.9	3.9 (2.2–6.8)
Mignard, 1986 [23]	Africa	Eastern Africa	242	385	45	18.6	32	8.3	96.9	2.5 (1.6–4.1)
Dumas, 1989 [24]	Africa	Western Africa	88	1439	27	30.7	98	6.8	100	6.1 (3.7–10.0)
Gracia, 1990 [25]	America	Central America	19	36	1	5.3	1	2.8	6.6	1.9 (0.1–32.9)
Dansey, 1992 [26]	Africa	South Africa	165	138	63	38.2	20	14.5	99.5	3.6 (2.1–6.4)
Nzisabira, 1992 [27]	Africa	Eastern Africa	98	30	40	40.8	1	3.3	96.9	20.0 (2.6–152.9)
Sarti, 1992 [28]	America	North America	16	1533	5	31.3	162	10.6	67.8	3.9 (1.3–11.2)
Garcia, 1993 [29]	America	South America	2667	750	108	4.1	16	2.1	66.4	5.0 (2.2–11.4)
Kong, 1993 [30]	Asia	Eastern Asia	189	309	22	11.6	8	2.6	98.6	1.9 (1.1–3.3)
Bouteille, 1994 [31]	Africa	Western Africa	22	1421	2	9.1	55	3.9	21.2	2.5 (0.6–10.9)
Theis, 1994 [32]	Asia	South-Eastern Asia	74	746	10	13.5	94	12.6	4.5	1.1 (0.5–2.2)
Aranda-Alvarez, 1995 [33]	America	North America	46	854	3	6.5	6	0.7	92.7	9.9 (2.4–40.8)
Grill, 1996 [34]	Africa	Eastern Africa	256	113	153	59.8	30	26.5	100	4.1 (2.5–6.7)
Andriantsimahavandy, 1997 [8]	Africa	Eastern Africa	104	104	33	31.7	14	13.5	88.6	3.0 (1.5–6.0)
Handali, 1997 [35]	Asia	South-Eastern Asia	241	260	163	67.6	215	82.7	99.2	0.4 (0.3–0.7)
Newell, 1997 [12]	Africa	Eastern Africa	103	72	12	11.7	2	2.8	56.8	4.6 (1.0–21.3)
Cruz, 1999 [37]	America	South America	26	118	14	53.8	17	14.4	99.4	7.0 (2.7–17.5)
Correa, 1999 [36]	America	North America	68	133	15	22.1	17	12.8	38	1.9 (0.9–4.2)
Balogou, 2000 [38]	Africa	Western Africa	115	1343	12	10.4	37	2.8	99.1	4.1 (2.1–8.1)
Mittal, 2001 [39]	Asia	Southern Asia	1881	50	196	10.4	1	2	96.1	5.7 (0.8–41.5)
Nicoletti, 2002 [13]	America	South America	113	233	22	19.5	27	11.6	47	1.8 (1.0–3.4)
Macharia, 2002 [40]	Africa	Eastern Africa	99	124	5	5.1	3	2.4	19.1	2.2 (0.5–9.2)
Rakatobe, 2002 [41]	Africa	Eastern Africa	99	107	2	2	13	12.1	72.4	0.2 (0.1–0.7)
Nsengiyumva, 2003 [6]	Africa	Eastern Africa	324	648	193	59.6	204	31.5	100	3.2 (2.4–4.2)
Dongmo, 2004 [42]	Africa	Middle Africa	93	81	17	18.3	12	14.8	9.3	1.3 (0.6–2.9)
Del Brutto, 2005 [43]	America	South America	19	19	5	26.3	1	5.3	42.8	6.4 (0.7–61.5)
Montano, 2005 [44]	America	South America	39	111	15	38.5	26	23.4	41.8	2.0 (0.9–4.5)
Li, 2006 [45]	Asia	Eastern Asia	55	445	9	16.4	11	2.5	99.9	7.7 (3.0–19.6)
Tran, 2007 [46]	Asia	South-Eastern Asia	31	124	1	3.2	6	4.8	5.2	0.7 (0.1–5.7)
Prasad, 2008 [47]	Asia	Southern Asia	60	107	29	48.3	31	28.9	70.9	2.3 (1.2–4.4)
Winkler, 2009 [10]	Africa	Eastern Africa	212	198	38	17.9	10	5.1	98.2	4.1 (2.0–8.5)
Secka, 2010 [48]	Africa	Western Africa	210	420	1	0.5	1	0.2	6.6	2.0 (0.1–32.2)
Nitiéma, 2012 [49]	Africa	Western Africa	39	814	5	12.9	28	3.4	67.1	4.1 (1.5–11.4)
Singh, 2012 [50]	Asia	Southern Asia	106	106	27	25.5	13	12	77.7	2.4 (1.2–5.1)
Elliott, 2013 [51]	Africa	Middle Africa	249	245	11	4.4	13	53	7.6	0.8 (0.4–1.9)
Ngugi, 2013 [52]	Africa	Eastern Africa	533	835	15	28.1	18	21.5	52.7	1.3 (0.7–2.6)

*Result = 0 in the study, modified to calculate the odds ratio, otherwise OR independent, SP: a posteriori statistical power

OR: Odds ratio, PWE + CC: people with epilepsy affected by cysticercosis, PWE—CC: people with epilepsy not affected by cysticercosis,

PWOE + CC: people without epilepsy affected by cysticercosis, PWOE–CC: people without epilepsy unaffected by cysticercosis

Overall, the global OR from 37 studies was estimated to be 2.7 with a 95% confidence interval of 2.1 to 3.6. This degree of association conforms to individual studies conducted elsewhere [8]. Another review from SSA yielded an OR of 3.4 [7].

Although we did not conduct any analyse based on the type of epilepsy, the literature suggests, although again not without exceptions, a stronger association of CC with late-onset epilepsy and partial seizures[4]. Another issue that can be raised is the temporality. We cannot be sure if seizures actually predated infection as several of our studies (see above) were cross-sectional surveys. Given the challenges in the availability of reliable patient records in most LMICs and excessive reliance on backward patient reporting about exposures to risk factors, even within case-control studies, it is not always and possible to confidently assess the temporality of this exposure before epilepsy becomes visible. [19].

Two different serological tests to detect *antibodies T solium* in serum were applied in 27/37 (72.97%) studies. In field conditions, EITB-LLGP (known as western blot or immunoblot) is a useful tool to identify exposure, but does not discriminate between active or inactive lesions. In the clinical setting, a positive EITB-LLGP can support a diagnosis of NCC when there are suggestive images on brain CT scan or MRI. The sensitivity of this test is reported to be 98% with 100% specificity [20]; however, the sensitivity is much lower for NCC with less than 2 parenchymal cysts or for calcified NCC. This is contrast to ELISA, which is specific to viable cyst infection (93.7%) but much less sensitivity in single-lesion[53]. The prevalence of viable NCC cases are low in field conditions (most of them asymptomatic) making this tool unhelpful for epidemiological interventions. Detection of CC would, therefore, depend on the type, accuracy, cost and availability of these tests. Studies that used EITB antibody detection gave a common OR of 1.9, much lower than the global OR obtained by taking into account all 37 studies. Other factors may also reduce the strength of any association between serologically-defined CC and other disease conditions, including *a)* high background seroprevalence in the general population (usually considered to be 10–25%), and *b)* many individuals with calcified CC become seronegative over the years [5].

The gold standard tool for determining CC exposure is to demonstrate the parasite in the CNS, by biopsy, although this is not without risk. Modern neuroimaging can provide strong evidence of NCC and should be done for both cases and controls. As shown above, many studies do not include neuroimaging due to cost, radiation exposure, and guidelines. Of the 37 studies reviewed, only eight used CT in both cases and controls; in these, the common OR reached 3.2, a value close to, but higher than, that obtained by considering all studies.

Based on our 37 studies, the etiologic fraction was estimated to be 63% among the exposed group in the population. This indicates an excellent opportunity to prevent a large fraction of late onset epilepsy given that CC can be prevented by controlling transmission of T. solium [21]. This study suggesst that adequate control measures and surveillance of CC in endemic regions should be key issues in preventing late-onset epilepsy in tropical regions.

### Perspectives

We propose that future field interventions should meet basic requirements to be more useful:

- Adequate design and use of validated surveys in community-based studies
- Case-control studies with high levels of exposure to CC
- Sufficient statistical power by recruiting adequate numbers of people with epilepsy and controls
- Matching of controls by sex, age and location
- Computed tomography of the brain without contrast and serological assays (Ag-ELISA and EITB) should be performed for all included subjects.
- Use of International League Against Epilepsy guidelines for epidemiological studies to standardized concepts of classification of epilepsy (ILAE 1993).
- Include family trees to assess familial history of seizures.
- Efforts should be made to assess all other possible risk factors for epilepsy.

## Conclusions

Cysticercosis is an active helminthic infection common in tropical regions. Many questions are still unanswered and there are still many limitations in epidemiological base-studies. Based on the current data, NCC is significantly associated with symptomatic epilepsy in low and middle-income countries. However, the strength of this association certainly varies depending on the transmission intensity (rural areas, poor sanitation, lack of potable water, etc). More meta-analyses that are meaningful require good quality studies in tropical regions following certain basic methodological requirements listed above. Finally, epilepsy attributable to CC is preventable. There is a need to focus our efforts on research, control and prevention of CC to avoid increased costly neurological morbidity of this zoonotic disease.

## Supporting information

S1 Checklist PRISMA checklist. (PDF)
